# Maternal Roux-en-Y gastric bypass surgery reduces lipid deposition and increases UCP1 expression in the brown adipose tissue of male offspring

**DOI:** 10.1038/s41598-020-80104-8

**Published:** 2021-01-13

**Authors:** Vanessa Marieli Ceglarek, Iala Milene Bertasso, Carla Bruna Pietrobon, Sofia Pizzato Scomazzon, Nayara Carvalho Leite, Maria Lúcia Bonfleur, Allan Cezar Faria Araújo, Sandra Lucinei Balbo, Sabrina Grassiolli

**Affiliations:** 1 Laboratory of Endocrine and Metabolic Physiology, Biosciences and Health, Postgraduate, University of West Parana, Cascavel, PR Brazil; 2 Medical Sciences: Endocrinology Post Graduate Program, Federal University of Rio Grande do Sul, Porto Alegre, RS Brazil; 3 Obesity Comorbidities and Research Center, University of Campinas, Campinas, SP Brazil; 4 Institute of Basic Health Sciences. Biological Sciences: Physiology, postgraduate. Department of Physiology, Room 337-7, Laboratory of Neurophysiology of Cognition and Development of the Brain, Federal University of Rio Grande do Sul, 500, Sarmento Leite - Farroupilha, Porto Alegre, RS 90050-170 Brazil

**Keywords:** Biological techniques, Cell biology, Molecular biology, Physiology, Endocrinology

## Abstract

Maternal obesity induced by cafeteria diet (CAF) predisposes offspring to obesity and metabolic diseases, events that could be avoided by maternal bariatric surgery (BS). Herein we evaluated whether maternal BS is able to modulate brown adipose tissue (BAT) morphology and function in adult male rats born from obese female rats submitted to Roux-en-Y gastric bypass (RYGB). For this, adult male rat offspring were obtained from female rats that consumed standard diet (CTL), or CAF diet, and were submitted to simulated operation or RYGB. Analysis of offspring showed that, at 120 days of life, the maternal CAF diet induced adiposity and decreased the expression of mitochondrial Complex I (CI) and Complex III (CIII) in the BAT, resulting in higher accumulation of lipids than in BAT from offspring of CTL dams. Moreover, maternal RYGB increased UCP1 expression and prevented excessive deposition of lipids in the BAT of adult male offspring rats. However, maternal RYGB failed to reverse the effects of maternal diet on CI and CIII expression. Thus, maternal CAF promotes higher lipid deposition in the BAT of offspring, contributing to elevated adiposity. Maternal RYGB prevented obesity in offspring, probably by increasing the expression of UCP1.

## Introduction

Nutritional and hormonal insults occurring during critical developmental periods, especially pregnancy and lactation, seem to explain the Developmental Origins of Health and Disease (DOHaD), particularly the high incidence of non-communicable diseases (NCDs), such as diabetes and cardiovascular diseases in adult life^1–4^. These early stages of development are important periods of vulnerability to nutritional, hormonal or stressor insults, since they are associated with intense cellular proliferation and differentiation, resulting in rapid changes in physiology, morphology and anatomy, which can persist into adulthood^5–8^.

Maternal obesity exerts a recognized impact on foetal development and on the postnatal stages and has significant metabolic effects on offspring. Worryingly, excess of white adipose tissue (WAT) and metabolic dysfunctions have often been observed in women during the reproductive period, including during the gestation and lactation periods, representing a risk factor for the health of the offspring. In this regard, maternal obesity is related to excessive visceral WAT, dyslipidaemia and glucose intolerance in offspring during adult life^1–4,9^. Interestingly, maternal obesity can also program the brown adipose tissue (BAT) of offspring, resulting in reduced thermogenesis and higher fat accumulation^10–12^, resulting in lower energy expenditure, and contributing to obesity installation in adulthood.

Bariatric surgery (BS), especially the mixed (restrictive and malabsorptive) Roux-en-Y Gastric Bypass (RYGB) technique, has been identified as the most efficient method for reducing body weight^13^. Evidences suggests that RYGB increases energy expenditure in humans^14–17^ and particularly in rodent models^18–22^, an event that may involve increased thermogenesis in BAT^19,22–25^. As a result, many obese women of reproductive age resort to BS and, surprisingly, this strategy affects the health of the descendant. In this regard, children born after maternal BS (gastrointestinal bypass surgery or biliopancreatic diversion), present smaller adiposity and improved lipid profile compared to brothers born before BS, with persistent effect throughout life^26,27^. Also, the offspring born after maternal RYGB are smaller, with less weight and lower fat compared to non-bariatric mothers^28^. However, the long-term consequences of maternal BS, especially RYGB, are poorly understood and its effects on the BAT of the offspring are unknown.

Reflecting its functional role, the BAT is characterized by a multilocular profile of lipid deposition, a high quantity of mitochondria, dense vascularization and innervations, being a thermogenic specialized tissue^29^. The mitochondrial inner membrane of the BAT adipocyte presents a high expression of uncoupling protein 1 (UCP1). The UCP1 generates and dissipates heat in a process known as thermogenesis. This process is a result of a proton gradient, due to oxidative phosphorylation (OXPHOS), mediated by the Electron Transport Chain (ETC): complexes NADH—Ubiquinone Oxidoreductase (CI), Ubiquinol–Cytochrome c Oxidoreductase (CIII) and Cytochrome c oxidase (CIV). Despite not being exclusive, the lipids beta-oxidation is a central energetic substrate used to sustain the respiratory chain in BAT. In this regard, lipids are made available through lipolysis, a process stimulated by norepinephrine (NE) released from the Sympathetic Nervous System (SNS), which richly innervates BAT adipocytes^30–32^. Thus, histological aspects, as well as lipid content and UCP1 expression, negatively and positively correlate with BAT thermogenesis, respectively^33^. However, maternal malnutrition and obesity can program the BAT of offspring, contributing to the disruption in energy homeostasis^10–12^.

The obesity is a condition frequently found in women of reproductive age, having deleterious effects on the health of offspring at long term, including negative impact in BAT thermogenesis process. The maternal BS is able to attenuate or avoid body weight gain and comorbidities in adult offspring. Considering, the central role of BAT in non-shivering thermogenesis and its impact in energy expenditure, the possibility of maternal BS modulates BAT function in offspring could not be discarded. Thus, in the present study, we aimed to analyse whether maternal RYGB promotes changes in the offspring's BAT. For this, we analysed morphological profile of lipids deposition and proteins expressions involved in thermogenic process [UCP1 and the ETC complex (CI- CV)] in BAT from male adult rats born from obese dams submitted to RYGB.

## Results

### RYGB surgery avoids excessive body weight gain, adiposity and disruption in glucose homeostasis in dams

**Figure 1:**
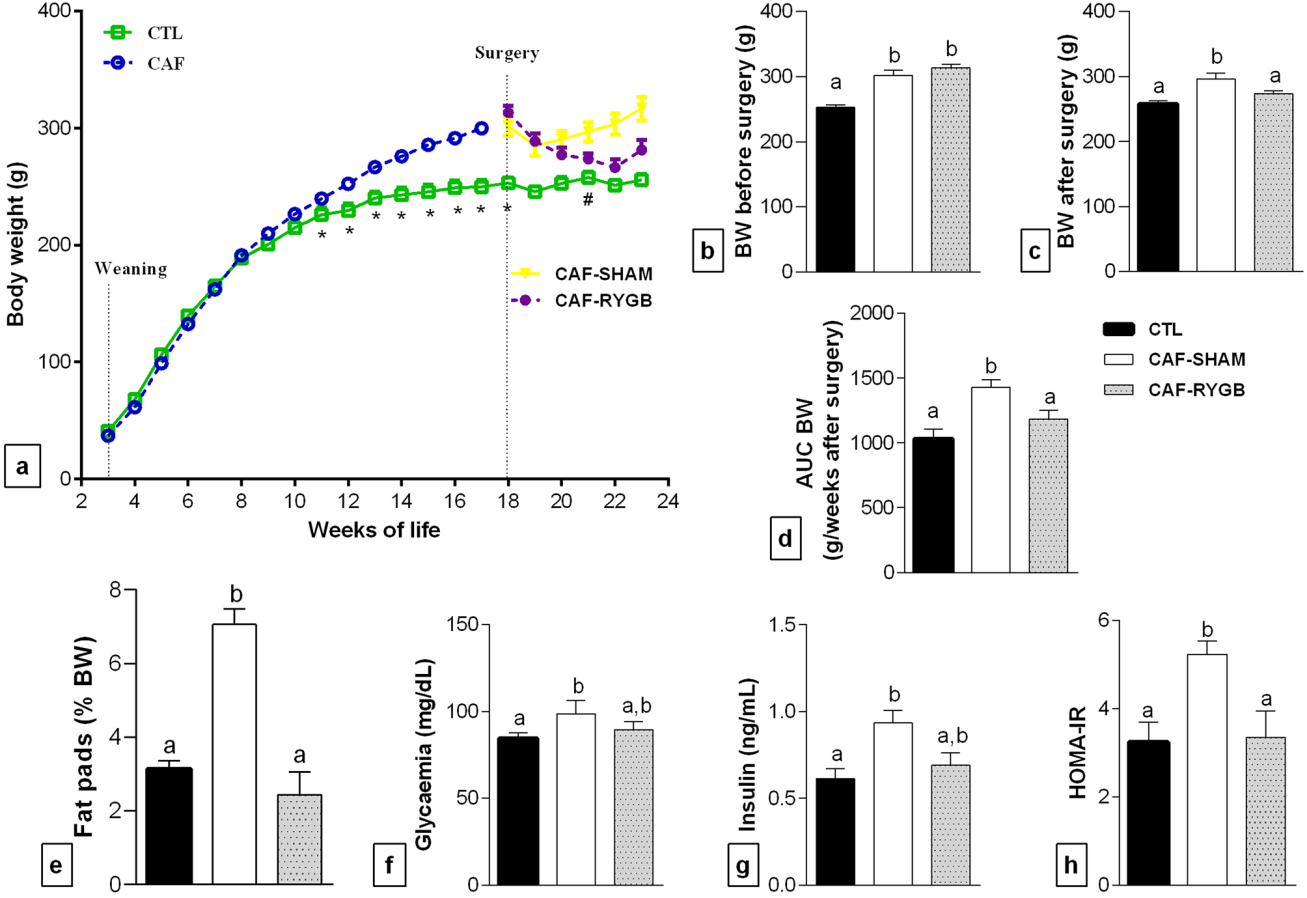
Biometric and serum biochemical parameters in maternal groups submitted, or not, to RYGB. CTL, female rats fed standard diet (n = 5–11 animals); CAF-SHAM, female rats fed on CAF diet and submitted to sham operation (n = 5–12 animals); CAF-RYGB, female rats fed on CAF diet and submitted to RYGB (n = 5–22 animals). Maternal biometric and biochemical parameters were analysed: Evolution of body weight with the CAF diet, before and after RYGB or SHAM operation (**a**), body weight before surgery (**b**), body weight after surgery (**c**), body weight area under curve (**d**), Fat pads (**e**), glycaemia (**f**), insulin (**g**), HOMA-IR (**h**). Different letters indicate statistical differences by one-way ANOVA and Bonferroni post-test (*p* < 0.05). The data are mean ± SEM. *p < 0.05 (Student’s t test). #*p* < 0.05 (CAF-RYGB vs CAF-SHAM).

Confirming efficacy of CAF diet intake, the body weight (BW) of female rats increased continuously from the eighth week receiving the CAF diet until RYGB or SHAM operation compared to the CTL group (Fig. 1a; *p* < 0.05). At surgery moment (18 weeks old), the CAF diet groups continued to have a higher BW than the CTL group [Fig. 1b; F_(2, 40)_ = 21.40, *p* < 0.0001]. Three weeks after surgery procedures, the female in the CAF-SHAM group maintained elevated BW compared to CTL animals. In contrast, the CAF-RYGB females, despite maintenance on CAF diet, showed significant reduction in BW with values similar to CTL female rats [Fig. 1c; F_(2,39)_ = 9.282, *p* = 0.0005]. This response was persistent in CAF-RYGB female throughout the experiment, as demonstrated by smaller area under curve (AUC) of BW compared to CAF-SHAM rats [Fig. 1d; F_(2,38)_ = 6.097, *p* = 0.0051]. At the time of euthanasia, female in the CAF-SHAM group showed excessive adiposity compared to CTL female, confirming obesity in this group. On the other hand, CAF-RYGB female rats had adipose tissue content similar to those observed in the CTL group [Fig. 1e; F_(2,16)_ = 37.04, *p* < 0.0001].

Higher adiposity in CAF-SHAM group was associated with metabolic abnormalities. Thus, CAF-SHAM dams had hyperglycaemia [Fig. 1f; F_(2,27)_ = 4.26, *p* = 0.0246], hyperinsulinemia [Fig. 1g; F_(2,12)_ = 6.106, *p* = 0.0148] and greater insulin resistance [Fig. 1h; F_(2,11)_ = 6.642, *p* = 0.0128] compared to CTL dams. The maternal RYGB surgery attenuates the disruption in glucose homeostasis. Thus, CAF-RYGB dams had fasting glucose and insulin plasmatic levels smaller than CAF-SHAM dams, but slightly elevated compared to CTL group, resulting in normalization of insulin sensitivity.

### Male offspring of CAF-RYGB female rats are born smaller and are growth-restricted during early postnatal life

Table 1 shows the effects of maternal RYGB on anthropometric and adiposity parameters of offspring (F1). The maternal CAF diet did not significantly alter the BW of offspring throughout life. Thus, the BW of the CAF-SHAM_F1_ group at birth [F_(2,21)_ = 12.422, *p* < 0.0001] weaning [(30 days) x^2^ = 13.396, *p* = 0.0012] and adulthood [(120 days) F_(2,21)_ = 7.936, *p* = 0.003] were similar to the BW of CTL_F1_ rats of the same ages. In contrast, the offspring in CAF-RYGB_F1_ group showed lower BW throughout life compared to CTL_F1_ and CAF-SHAM_F1_ groups. Additionally, CAF-RYGB_F1_ presented a reduction in naso-anal length (NAL), when compared to CTL_F1_ and CAF-SHAM_F1_, at 120 days of life [F_(2,21)_ = 7.357, *p* = 0.004]. The CAF maternal diet per se did not influence offspring growth. Thus, CTL_F1_ and CAF-SHAM_F1_ rats showed similar NAL at 120 days of life. Neither maternal CAF diet nor BS significantly affected Lee’s Index in offspring at 120 days of life [F_(2,21)_ = 1.121, *p* = 0.345].

**Table 1:** Effect of maternal obesity and RYGB on the anthropometric parameters of male adult offspring rats.

Parameters	CTL_F1_	CAF-SHAM_F1_	CAF-RYGB_F1_	p-value
BW at birth (g)	0.012 ± 0.0006^a^	0.013 ± 0.001^a^	0.007 ± 0.0004^b^	0.0001
BW at weaning (g)#	36.5 [31.0 – 67.0]^a^	42.5 [34.0 – 58.0]^a^	23.0 [18.0 – 36.0]^b^	0.049
BW at adulthood (g)	373.6 ± 9.03^a^	379.8 ± 18.64^a^	303.7 ± 18.15^b^	0.003
NAL (cm)	22.63 ± 0.17^a^	22.67 ± 0.33^a^	21.42 ± 0.30^b^	0.004
Lee Index	0.318 ± 0.002^a^	0.319 ± 0.003^a^	0.314 ± 0.002^a^	0.345
WAT-R (g/100 g)	0.615 ± 0.06^a^	0.950 ± 0.14^b^	0.482 ± 0.07^a^	0.008

BW, body weight; NAL, naso-anal length; WAT-R, white adipose tissue- retroperitoneal; AUC, area under curve; g, gram; cm, centimetre. Offspring (_F1_): CTL_F1_: Offspring from dams that consumed rodent chow diet throughout life and were not operated; CAF-SHAM_F1_: Offspring from dams that consumed CAF diet throughout life and were submitted to SHAM surgery; CAF-RYGB_F1_: Offspring from dams that consumed CAF diet throughout life and were submitted to RYGB (n = 6 rats/groups). Data are means ± SEM. Different letters represent statistical differences between the groups. One-way ANOVA, Bonferroni post-test (p < 0.05). # Kruskall-Wallis, Dunn's test (p < 0.05), median and 25th and 75th percentiles.

The maternal CAF diet caused adiposity in adult offspring, and this adiposity was modified by maternal RYGB. At 120 days of life, the offspring of the CAF-SHAM_F1_ group presented a higher WAT-R content than the CTL_F1_ offspring_,_ while the offspring of female rats submitted to the RYGB showed a reduction in WAT-R, compared to offspring of non-operated female rats, resembling the CTL_F1_ group [F_(2,21)_ = 6.14, *p* = 0.008].

### Maternal RYGB surgery changes food intake, body weight gain and energy expenditure of adult male offspring

**Figure 2:**
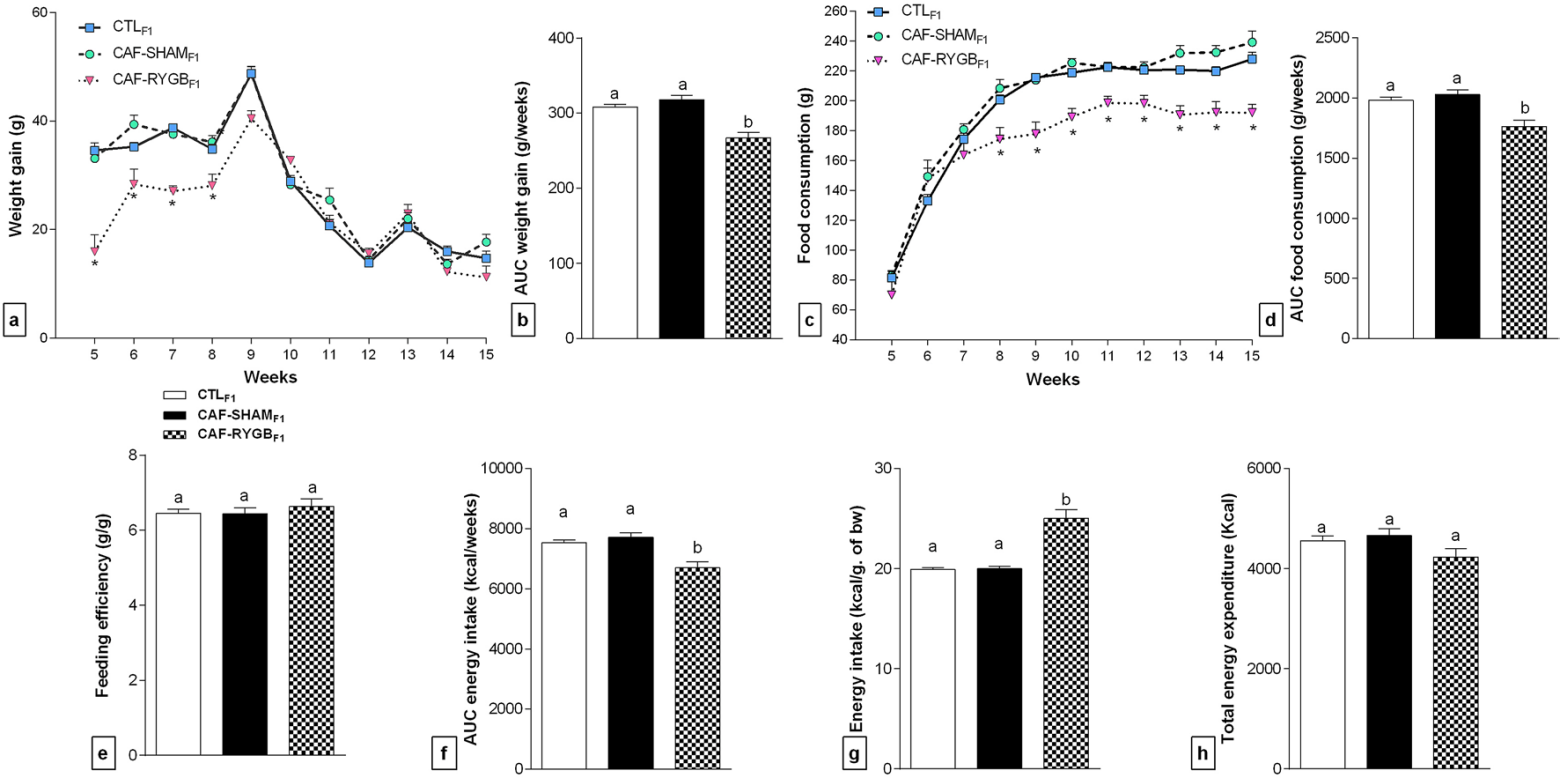
Metabolic parameters in offspring from dams submitted, or not, to RYGB. CTL_F1_, offspring of rats fed standard diet (n = 36 animals); CAF-SHAM_F1_, offspring of rats fed on CAF diet and submitted to sham operation (n = 28 animals); CAF-RYGB_F1_, offspring of rats fed on CAF diet and submitted to RYGB (n = 14 animals). Weight gain (**a**) and food intake (**c**) were measured weekly. Metabolic parameters were analysed and presented, by AUC, the weight gain (**b**), total food consumption (**d**), feeding efficiency (**e**), total energy intake (**f**), energy intake per gram of body weight (**g**) and total energy expenditure (**h**). Different letters indicate statistical differences by one-way ANOVA and Bonferroni post-test (*p* < 0.05). The data are mean ± SEM. *p < 0.05.

Throughout development, neither BW gain (Fig. 2a,b) nor food consumption (Fig. 2c,d) were significantly modified in the CAF-SHAM_F1_ rats compared to CTL_F1_ animals. Thus, the feeding efficiency (Fig. 2e), the absolute (Fig. 2f) and relative energy intake (Fig. 2g), as well as, the energy expenditure (Fig. 2h) were similar between CAF-SHAM_F1_ and to CTL_F1_.

In contrast, a dual profile of BW gain was observed in CAF-RYGB_F1_ group throughout life (Fig. 2a). Thus, from 5 to 9th week of life, the CAF-RYGB_F1_ rats showed reduced BW gain [5th week: F_(2, 75)_ = 18.6, *p* < 0.0001; 6th week: F_(2, 72)_ = 9.53, *p* = 0.0002; 7th week: F_(2, 75)_ = 33.88, *p* < 0. 0001; 8th week: F_(2, 75)_ = 8.507, *p* = 0.0005; 9th week: F_(2, 75)_ = 7.700, *p* = 0.0009], compared to CTL_F1_ and CAF-SHAM_F1_ groups. Meanwhile, from 10 to 15th week of life, the BW gain in the CAF-RYGB_F1_ rats was similar to CTL_F1_ and CAF-SHAM_F1_ groups (*p* > 0.05). Nevertheless, the AUC of BW gain throughout life was lower in CAF-RYGB_F1_ group compared to both, CTL_F1_ and CAF-SHAM_F1_ groups [Fig. 2b F_(2, 75)_ = 16.91, *p* < 0.0001].

Similarly, the food intake presented fluctuation throughout life in CAF-RYGB_F1_ group (Fig. 2c). Thus, the food intake was similar between CAF-RYGB_F1,_ CTL_F1_ and CAF-SHAM_F1_ groups from 5 to 7th weeks of life (*p* > 0.05). However, from 8 to 15th weeks of life, the CAF-RYGB_F1_ rats consumed less food compared to CTL_F1_ and CAF-SHAM_F1_ animals [Fig. 2c; 8th week: F_(2, 75)_ = 8.445, *p* = 0.0005; 9th week: F_(2, 75)_ = 21.39, *p* < 0.0001; 10th week: F_(2, 75)_ = 22.59, *p* < 0. 0001; 11th week: F_(2, 75)_ = 18.72, *p* < 0.0001; 12th week: F_(2, 75)_ = 6.11, *p* = 0.0035; 13th week: F_(2, 75)_ = 17.17, *p* < 0.0001; 14th week: F_(2, 75)_ = 3.649, *p* = 0.0307; 15th week: F_(2, 75)_ = 5.302, *p* = 0.0070].

In consequence, the total AUC of food consumption (Fig. 2d) and total AUC of energy intake (Fig. 2f) were lower in the CAF-RYGB_F1_ rats compared to CTL_F1_ and CAF-SHAM_F1_ groups (x^2^ = 12.07, *p* < 0.0024). However, CAF-RYGB_F1_ group showed higher energy intake (kcal/g) [Fig. 2g; F_(2, 74)_ = 47.34, *p* = 0.00001] compared to CTL_F1_ and CAF-SHAM_F1_ groups. Despite this, CAF-RYGB_F1_ group did not present significant alteration in feeding efficiency (Fig. 2e; x^2^ = 1.009, *p* = 0.6039) and total energy expenditure [Fig. 2h; F_(2, 74)_ = 2.035, *p* = 0.1379].

### Effects of maternal CAF diet and RYGB on BAT of adult male offspring

**Figure 3:**
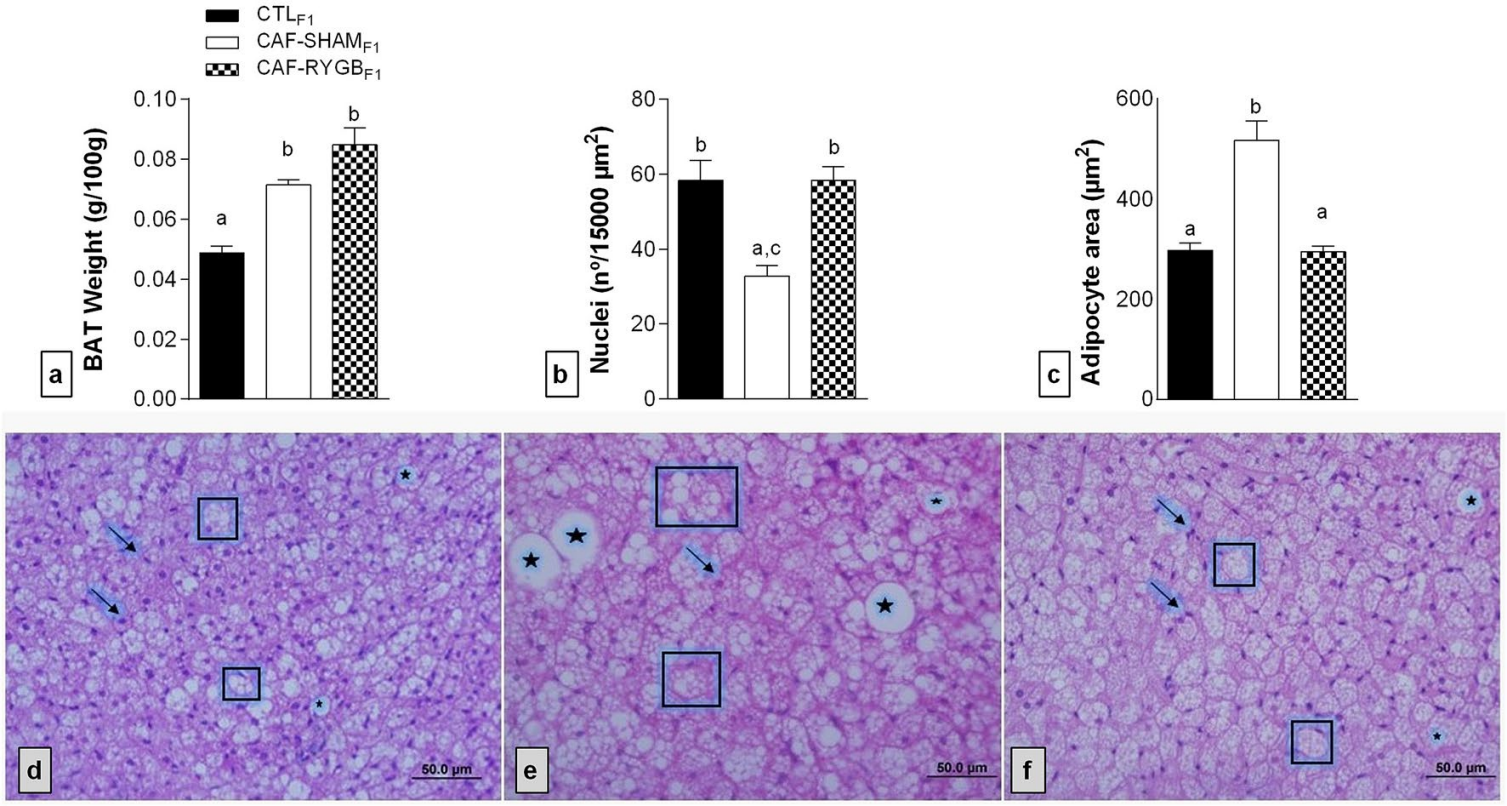
Effect of maternal obesity and RYGB on BAT weight and the nucleus number and adipocyte size in the BAT of male adult offspring rats. CTL_F1_, offspring of rats fed standard diet; CAF-SHAM_F1_, offspring of rats fed on CAF diet and submitted to sham operation; CAF-RYGB_F1_, offspring of rats fed on CAF diet and submitted to RYGB (n = 6 animals/group). Weight of BAT (**a**), nucleus number (**b**) and adipocyte size (**c**) were analysed by light microscopy. Different letters indicate statistical differences by one-way ANOVA and Bonferroni post-test (*p* < 0.05). Representative photomicrograph (insert) of BAT (40x), stained with H&E are showed in the figures (**d**) (CTL_F1_), (**e**) (CAF-SHAM _F1_) and (**f**) (CAF-RYGB_F1_). Arrows indicate nuclei and boxes indicate adipocyte area. The data are mean ± SEM.

The maternal CAF diet increased the fat deposition in the BAT of male adult offspring, while maternal BS prevented this accumulation of fat (Fig. 3). BAT weight was higher in animals of the CAF-SHAM_F1_ and CAF-RYGB_F1_ groups compared to the BAT of the CTL_F1_ group [Fig. 3a; F_(2,14)_ = 7.519, *p* < 0.0001]. In CAF-SHAM_F1_ rats_,_ reduction in nucleus number (Fig. 3b) and increased adipocyte size (Fig. 3c) was observed compared to the BAT of the CTL_F1_ group. Conversely, BS abolished these changes in the BAT of CAF-RYGB_F1_ rats (Fig. 3f), which presented an increase in the number of nuclei [Fig. 3b; F_(2,15)_ = 13.293, *p* < 0.001] and a reduction in adipocyte size [Fig. 3c; F_(2,15)_ = 27.56, *p* < 0.001], compared to CAF-SHAM_F1_, resembling the CTL_F1_ group.

**Figure 4:**
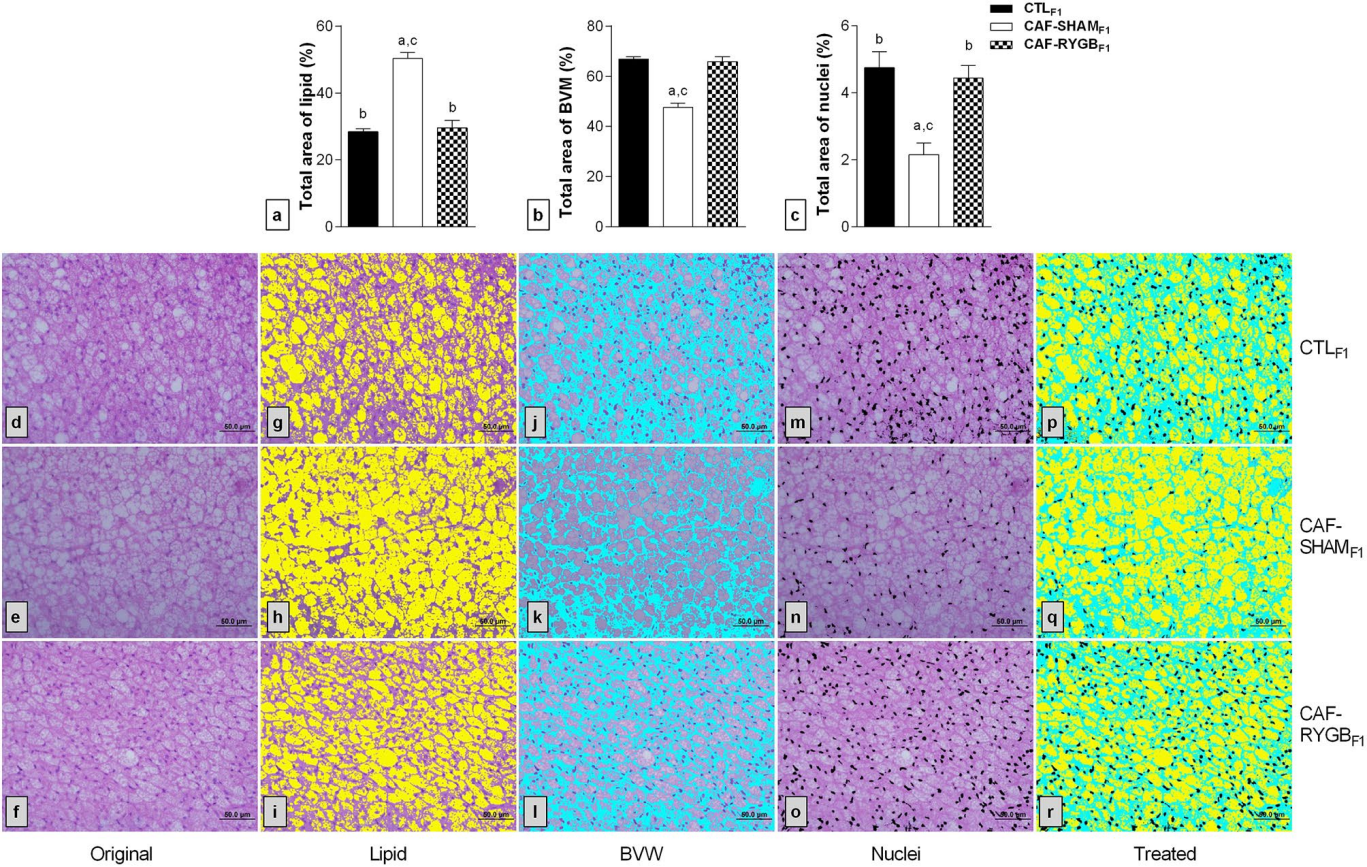
Effect of maternal obesity and RYGB on the distribution of fat, nuclei and BVM in the BAT of male adult offspring rats. CTL_F1_, offspring of rats fed standard diet; CAF-SHAM_F1_, offspring of rats fed on CAF diet and submitted to sham operation; CAF-RYGB_F1_, offspring of rats fed on CAF diet and submitted to RYGB (n = 6 animals/group). The percentages of the total area occupied by lipids (**a**), BVM (**b**) and nuclei (**c**) were analysed. Different letters indicate statistical differences by one-way ANOVA and Bonferroni post-test (*p* < 0.05). Representative photomicrograph (insert) of BAT (40x), stained with H&E are showed in the figures (**d**) (CTL_F1_), (**e**) (CAF-SHAM_F1_) and (**f**) (CAF-RYGB_F1_). The images were processed with Image J Program as explained in methods section. Groups are in rows. CTL_F1_, CAF-SHAM_F1_ and CAF-RYGB_F1._ In columns original image (**d**–**f**), adipocytes in yellow (**g**–**i**), BVM in blue (**j**–**l**) and Nuclei in black (**m**–**o**). Fully treated image (**p**–**r**). The data are means ± SEM.

As a result of these changes (Fig. 4), the fat area in the BAT of the CAF-SHAM_F1_ group (Fig. 4h) demonstrated an average of 51% of the total BAT area (Fig. 4a). In contrast, the fat in the BAT of CAF-RYGB_F1_ rats (Fig. 4i) was 30% of the area, similar to that of CTL_F1_ rats [28%; Fig. 4g; F_(2,15)_ = 54.23, *p* < 0.001]. Consequently, the percentage of BVM (Fig. 4b) and nuclei (Fig. 4c) decreased in the BAT of CAF-SHAM_F1_ rats to 47% (Fig. 4k) and 2% (Fig. 4n), respectively. Thus, the percentages of BVM (66%, Fig. 4l) and nuclei (4%, Fig. 4o) in the BAT of CAF-RYGB_F1_ rats were similar to the percentages of BVM (67%, Fig. 4j) and nuclei (5%, Fig. 4m) in the CTL_F1_ group [F_(2,15)_ = 46.036, *p* < 0.0001 and F_(2,15)_ = 9.226, *p* < 0.001, respectively].

**Figure 5:**
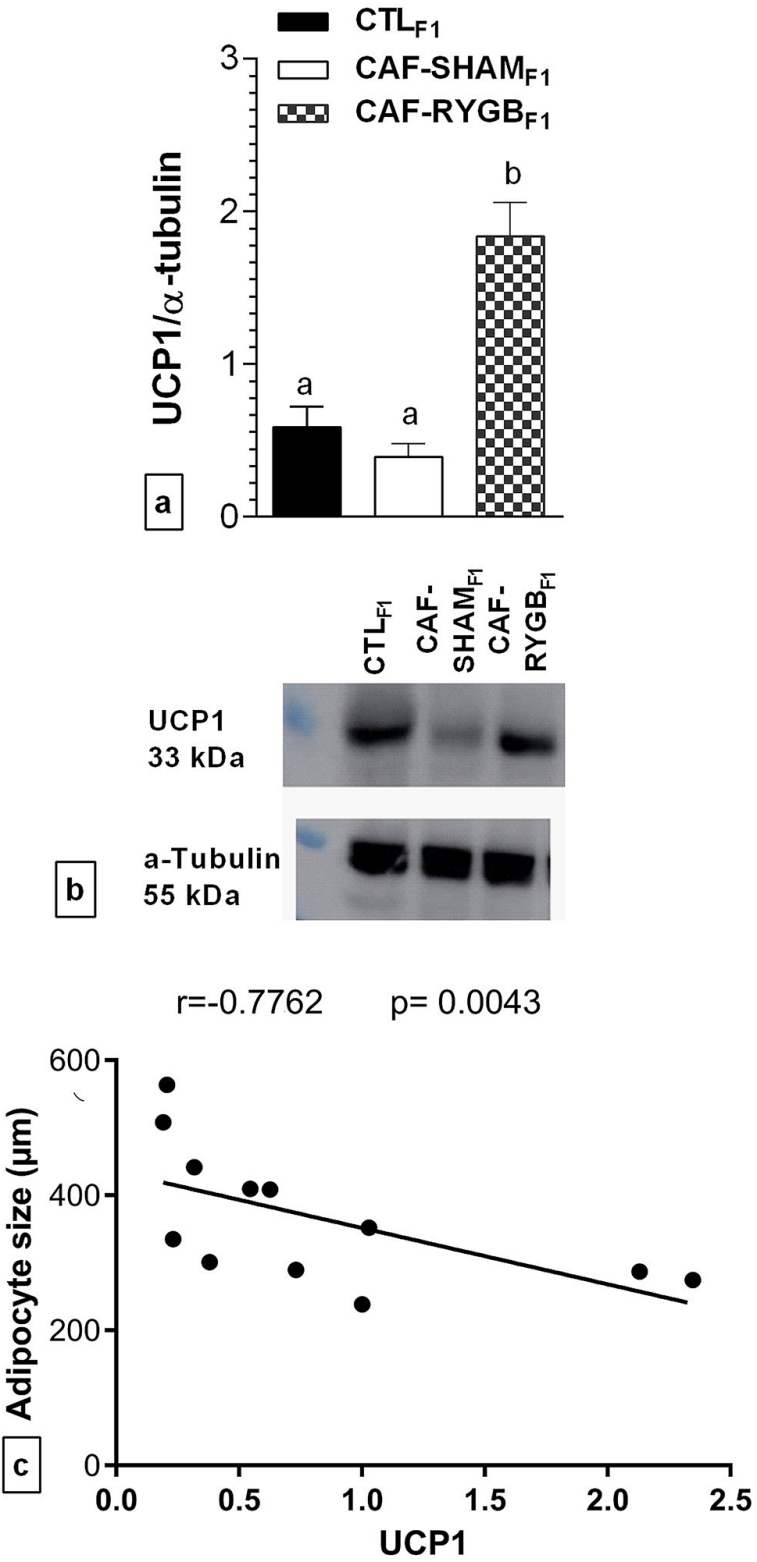
Effect of maternal obesity and RYGB on UCP1 expression in BAT of adult male offspring. CTL_F1_, offspring of rats fed standard diet; CAF-SHAM_F1_, offspring of rats fed on CAF diet and submitted to sham operation; CAF-RYGB_F1_, offspring of rats fed on CAF diet and submitted to RYGB (n = 4 animals/group). UCP1 expression (**a**), was analysed by Western Blot, normalized by α tubulin protein expression (**b**), and correlated UCP1 expression with adipocyte size (**c**). Data are represented as means ± SEM. Different letters indicate statistical differences by one-way ANOVA and Bonferroni post-test (*p* < 0.05). UCP1, uncoupling protein 1. Correlation was analysed using nonparametric Spearman’s test. *p* values less than 0.05 were considered significant. Uncropped images in supplementary figure.

Maternal CAF diet did not alter the expression of UCP1 in offspring (Fig. 5). Although a 33% reduction in the expression of UCP1 was observed in the BAT of CAF-SHAM_F1_ rats, this expression was statistically similar to that of CTL_F1_ rats. On the other hand, maternal RYGB promoted changes in UCP1 expression. Thus, in the CAF-RYGB_F1_ group, the expression of UCP1 was increased by more than 300%, compared to the CTL_F1_ and CAF-SHAM_F1_ groups [Fig. 5a; F_(2,9)_ = 14.604, *p* = 0.001]. Of note, BAT adipocyte size showed an inverse correlation with the protein expression of UCP1 (Fig. 5b; r = − 0.7762, p = 0.0043).

**Figure 6:**
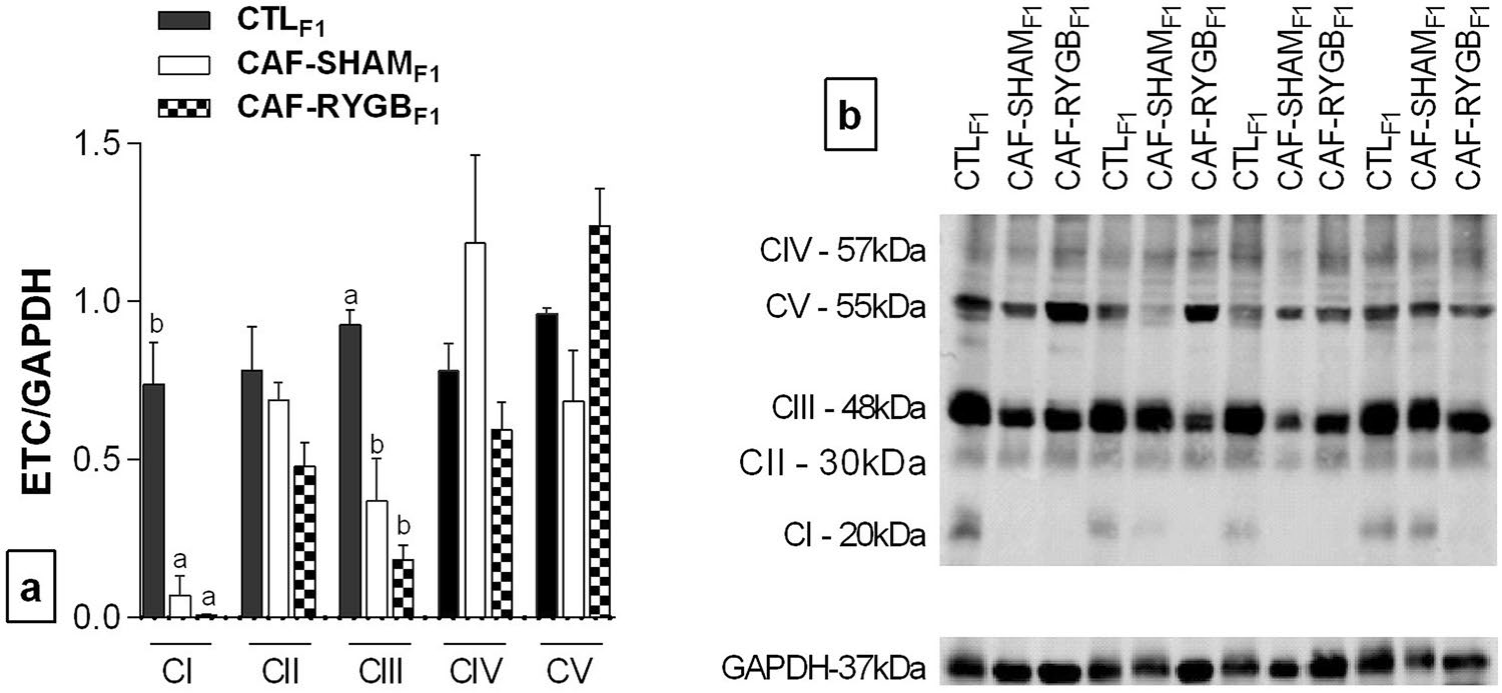
Effects of maternal obesity and RYGB on the expression of complexes (CI-CV) of the electron transport chain in adult offspring. CTL_F1_, offspring of rats fed standard diet; CAF-SHAM_F1_, offspring of rats fed on CAF diet and submitted to sham operation; CAF-RYGB_F1_, offspring of rats fed on CAF diet and submitted to RYGB (n = 4 animals/group). ETC protein expression (**a**) was analysed by Western Blot and normalized by GADPH protein expression (**b**). Different letters indicate statistical differences by one-way ANOVA and Bonferroni post-test (*p* < 0.05). ETC, electron transport chain. GAPDH, Glyceraldehyde 3-phosphate dehydrogenase. CI, CII, CIII, CIV, CV, complex I–V. Data are represented as means ± SEM. Uncropped images in supplementary figure.

The maternal CAF diet altered the protein expressions of the CI and CIII complexes in the BAT of male adult offspring rats and maternal BS did not modify this effect (Fig. 6). Accordingly, a reduction of more than 85% in the expression of CI was observed in the BAT of the CAF-SHAM_F1_ and CAF-RYGB_F1_ groups, compared to the BAT of the CTL_F1_ animals [F_(2,9)_ = 17.99, *p* = 0.001]. Similarly, the protein expression of CIII was reduced by 50% in the BAT of the CAF-SHAM_F1_ and CAF-RYGB_F1_ rats, compared to the BAT of the CTL_F1_ groups [F_(2,9)_ = 22.039, *p* = 0.0001]. Although the mean expression of CIV was 50% and 75% higher in the CAF-SHAM_F1_ group, compared to the CTL_F1_ and CAF-RYGB_F1_ groups, respectively, this difference was not statistically significant [F_(2,9)_ = 2.918, *p* = 0.105]. With regard to CII [F_(2,9)_ = 0.659, *p* = 0.541] and CV expression [F_(2,9)_ = 0.178, *p* = 0.840], there were no statistical differences between the groups.

## Discussion

The DOHaD is the study of how the early developmental stages, such as pregnancy and lactation, and early life environment can impact the risk of chronic diseases from childhood to adulthood, and the mechanisms involved^5–8^. Here, in support of this hypothesis, we show that offspring of obese female rats fed on a CAF diet have high adiposity in adulthood. Similar findings were reported in a study that evaluated the offspring of female rats resistant to obesity that were fed western diet^34^. Data highlight the consequences of maternal diet on the adiposity of offspring and show that maternal CAF diet induces metabolic dysfunctions such as insulin resistance, glucose intolerance, dyslipidaemia, and hypertension, resulting in metabolic syndrome development^1–5,8,9^. These effects create a vicious cycle of obesity in the next generations, which may explain the prevalence of obesity in the world and suggest that this pathology may further increase in the coming decades.

Thermogenesis in BAT is important for energy expenditure and adequate body weight control. Thus, reduced thermogenesis, greater lipid deposition and lower UCP1 expression in BAT occur frequently in obese rodent models^35,36^. Moreover, maternal obesity also results in the metabolic programming of BAT in offspring, although contradictory results have been observed^10–12^. Our data showed that the maternal CAF diet, from conception to lactation and weaning, modulated the BAT of the adult offspring, resulting in high lipid deposition, with hypertrophy of adipocytes and reduced proliferation of nuclei. The lipids are the main energetic substrate for β-oxidation in BAT, a process directly stimulated by NE released from nerve terminals in the SNS^29^. Therefore, the reduction in β-oxidation decreases the magnitude of the proton gradient, resulting in lower UCP1 expression^29–31,37^ and BAT hypoactivity. Corroborating with these findings, our study showed direct relationship between the lower expression of UCP1 and augmented size of the adipocytes in BAT. Similar results, also were observed by Tarabra et al.^33^, suggesting that thermogenic function was affected. Although the reduction in UCP1, observed here in the BAT of offspring born from CAF dams was not statistically significant, this observation corroborates with a study that showed that maternal high-fat diet (HFD) during lactation reduces UCP1 expression in BAT of offspring, a response partly due to attenuation of cellular β3-adrenergic signalling^12^. Therefore, it is possible that the offspring of obese female rats have reduced lipolysis due to alteration SNS activity in BAT and, consequently, lower expression of UCP1 and thermogenesis.

In agreement with this hypothesis, in the present study, for the first time, we showed that maternal obesity, induced by the CAF diet, can program the ETC of BAT in offspring, resulting in reductions in the expressions of CI and CIII. Similarly, Yu et al.^38^ have demonstrated that offspring born from diabetic dams present smaller expression of ETC and UCP1 in the BAT due to epigenetic DNA methylation. These findings indicate that the offspring of obese dams have a reduced flow of energetic substrates for oxidative phosphorylation, including lipids, and consequently a lower magnitude of the proton gradient, which is associated with histological characteristics, such as greater lipid deposition, suggesting hypoactivity of thermogenesis. Our data are in line with the work of Wang et al.^39^, which showed that maternal obesity increases triglyceride content and white adipose markers in foetal BAT, suppressing myogenesis and brown adipogenesis. Thus, epigenetic changes occur as a consequence of maternal obesity-related factors with persistent effects in adulthood.

Epigenetic molecular mechanisms have been observed in obesity^40–42^ and some studies infer that BS could regulate or reprogram the epigenetic obese programming^41–44^, specially the RYGB^42,44^. The BS promote rapid weight loss and correct metabolic abnormalities common in obesity^13^, such as changing the molecular pathways involved in inflammatory and immunological response, cell differentiation, oxidative stress regulation^45^, significantly reduces visceral WAT content, restores insulin sensitivity and normalizes glucose and lipid homeostasis^46^. In addition, BS changes serum concentrations of branched-chain amino acids and fatty acids, urinary concentrations of microbial co-metabolites and components of the gut microbiota, and all these changes may have effects on pregnancy^47^. Thus, maternal BS could be a reprogramming strategy to reverse adverse metabolic effects of maternal obesity in descendants. In this regard, it has already been shown that children born from mothers submitted to different types of BS present a more adequate glycaemic and lipaemic profile and reduced adiposity, when compared to children born before the maternal BS procedure^26,27^.

Maternal BS, however, also promotes deleterious effects on health of offspring, such as nutritional vitamins deficiency, increased risk of prematurity, lower weight at birth^48^, insulin resistance, β-cell dysfunction^49^, and lipid homeostasis dysfunction^50^. In the present study, male rats born from dams undergoing RYGB demonstrated reduced BW at birth, reduced growth throughout life and lower adiposity in adulthood. Similar findings demonstrated that maternal RYGB determines smaller adiposity and body weight at birth, suggesting metabolic programming effect of BS on offspring^28^. The same were reported following vertical sleeve gastrectomy in obese female rats fed with HFD, which increased the rate of small-for-gestational age offspring in rats^50^, suggesting intrauterine growth restriction caused by BS.

RYGB technique, in obese female mice, restored hypothalamic pathways involved in the control of food intake^51^. This effect may be a consequence of the alterations, caused by RYGB, in the intestinal microbiota and microbial production of short-chain fatty acids (SCFAs)^52^. The SCFAs participate of the regulation of appetite^53^ and can modulate neuronal activity and visceral reflexes^54^. However, it is still unknown if maternal RYGB can also modify hypothalamus from offspring. Nevertheless, here we show that offspring born from RYGB dams showed an imbalance in food intake and body weight gain throughout life. Thus, we observed that maternal RYGB promoted resistance to BW gain early in the offspring's life, without altering the energy intake. However, this effect was reversed in the offspring's adult life, and the energy intake was reduced. Thereby, the total energy consumption in relation to BW (Kcal/g) was elevated in offspring from RYGB dams, suggesting dysfunction in the homeostatic energy control. Interestingly, Kimura et al.^55^ showed that during pregnancy the SCFAs from the maternal gut microbiota can influence prenatal development of the metabolic and neural systems, promoting maintenance of postnatal energy homeostasis and resistance to obesity in offspring mice. Thus, similar effects may have happened in the present study.

Regarding energy homeostasis, adult offspring born from obese female rats, in the present work, had higher lipids deposition in BAT associated with lower expression of UCP1, suggesting reduced thermogenesis. For the first time, we showed that maternal RYGB could avoid excessive lipid deposition in the BAT of offspring adult male rats, a response intimately related to the overexpression of UCP1. These data reinforce the study of Cannon and Nedergaard^29^, who showed that UCP1 is the most important protein for thermogenesis, where its activity and expression are directly related to the metabolic state and SNS activity. Thus, it is possible that adult male rats born from obese dams submitted to RYGB presented increased SNS activity and consequently higher BAT lipolysis rate. This can provide free fatty acids to stimulate UCP1 expression or activity, resulting in elevated energy expenditure due to acceleration of the thermogenesis process. Therefore, elevated thermogenesis in BAT from offspring of obese dams submitted to RYGB may explain the reduction in lipid area and increase in nuclei and BVM, as well as the reduction in visceral WAT content, as observed in the present study. However, considering that BS may have both beneficial and harmful effects on pregnancy, further studies are necessary to evaluate the benefits and harms of maternal BS in offspring.

Surprisingly, we also observed that the effects of maternal obesity on ETC of adult offspring appears to be irreversible. As demonstrated by our results, offspring born from obese female rats had significantly reduction in mitochondrial expression of CI and CIII on BAT, suggesting maternal programming in ETC. The CI and CIII of ETC are part of supercomplex I-III, which exerts central functions in oxidative phosphorylation^56,57^, but these complexes are often regarded as the major sites of mitochondrial reactive oxidative species (mtROS) production^58,59^. Thus, studies have shown that mice fed with HFD demonstrated low activity and expressions of the OXPHOS subunits in the liver^60^ and developed left ventricular hypertrophy, which was associated with a selective reduction in the activity of cardiac mitochondrial OXPHOS CI and CIII and increased malondialdehyde production, a marker of oxidative stress^61^.

Unexpectedly in our data, maternal RGYB was able to increase UCP1 expression in BAT of offspring but inefficient to revert the reduction of CI and CIII. Castillo et al.^62^ showed that the mitochondrial transcription termination factor-4 (MTERF4) absence in BAT leads to reduced OXPHOS mitochondrial protein levels and impaired assembly of OXPHOS CI, III and IV due to deficient translation of mitochondrial DNA-encoded proteins. MTERF4-FAT-KO mice showed low OXPHOS, reduced thermogenesis activity but no alteration in UCP1 protein expression. It is important to note that mitochondrial DNA appears to be primarily inherited only from mothers, and it has much bigger role on mitochondrial metabolism of descendants^63^. Thus, it is possible that mitochondrial genes in ETC may be definitively programmed by maternal CAF diet. Moreover, some studies demonstrate that the increase in UCP subtype 2 (UCP2), in other organs, occurs due to the increase in mitochondrial ROS signalling, which works as a protective mechanism against oxidative stress^60,64^. Interestingly, Dimova et al.^65^ have shown that in addition to promoting intrauterine growth restriction, intrauterine oxidative stress promotes increased energy expenditure due to browning of WAT, as indicated by higher UCP1 expression, and protects the offspring against diet-induced adiposity, insulin resistance and hyperlipidaemia. Although in our study we did not evaluate the browning of WAT, increased UCP1 expression on BAT of the offspring of RYGB female rats may constitute a mechanism to attempt to control mitochondrial ROS production and the development of pathologies, since BS did not re-establish the expressions of CI and CIII, and our group has already showed^49^ that maternal RYGB in rats can negatively affect the growth and insulin secretion of offspring, resulting in insulin resistance and β-cell dysfunction in adult life. Thus, these findings support those and previous studies demonstrating that obesity per se and obesity-associated pathologies are related to mitochondrial dysfunction, such as progression of aging in adipocyte mitochondria, diabetes ^66^ and Cushing's syndrome^67^. Moreover, it emphasizes the nutritional status of the mother on future diseases in offspring.

Despite the important data reported in the present study, limitations of the investigation should be pointed out. The RYGB is able to induce browning in WAT^21,68^. However, here only the impact of maternal RYGB in BAT was evaluated. The offspring in this study were fed on a regular rodent diet throughout life and, thus, were not exposed to a hypercaloric environment throughout development, as proposed by Gluckman^69^, in the predictive adaptive response. Moreover, BAT thermogenesis is more effective activated by stressor conditions, such as, cold or exercise, and these situations could demonstrate thermogenic dysfunction in BAT more clearly. In addition, BAT histological and functional aspects could be consequence of metabolic states of offspring. Thus, measures of BAT parameters in offspring should be done at several stages of life (weaning, adult and old). Finally, it is necessary to access if the changes in the BAT of the offspring are direct effects of maternal RYGB (data indicate that anthropometric parameters are results of RYGB per se^28^) or maternal weight loss (evidence suggests that this event enhances adipose tissue thermogenesis^70^).

In conclusion, in the present study, we demonstrated that maternal obesity can lead to BAT programming in offspring during adulthood, resulting in decreased expressions of CI and CIII in mitochondria, culminating in the excessive accumulation of lipids in the adipocyte of BAT, possibly mediated by reduced β-oxidation flow. This hypofunction of BAT probably contributes to the high adiposity found in the offspring of obese dams. The maternal RYGB technique effectively reduces adiposity in adult offspring, restoring the thermogenesis of BAT, by promoting overexpression of UCP1, increasing β-oxidation and reducing fat accumulation in BAT, leading to anthropometric alterations at birth and adulthood. Although initially the data seems to be beneficial, little is known about the consequences of these changes in the offspring and it is still possible that these changes can be reversed throughout life and the descendants could develop further pathologies. Thus, these data reinforce the need to further study on the programming effect of maternal bariatric surgery on offspring.

## Methods

This study was approved by the Ethics Committee of the University of West Parana in 13/02/2015. We evaluated the first generation of offspring (F1) obtained from earlier phases, as briefly described. At 21-day-old Wistar female rats were randomly divided into two experimental groups, according to the diet offered. The Control group (CTL; n = 13) received a rodent diet (BioBase, Brazil), which has 3.8 kcal/g (70% carbohydrate, 20% protein and 10% fat) and water ad libitum, as recommended by AIN93^71^. The Cafeteria group (CAF; n = 34) received a cafeteria diet, consisting of 5.4 kcal/g as described by Balbo et al.^72^ and available, with adaptation, in Supplementary Table 1. At 130 days of life, half of the female CAF group rats were submitted to RYGB, according to our previous description^49^, while the other half of the CAF group underwent simulated surgery.

### RYGB and SHAM operations

Briefly, for RYGB operation, the stomach was divided forming a gastric pouch (5% of total organ volume). Then, the jejunum was cut (10 cm after the ligament of Treitz) and the distal limb was connected to the gastric pouch. The proximal limb of the jejunum was reconnected downward at a distance of 15 cm from the gastrojejunostomy. For the SHAM operation, the stomach was exposed, and the small intestine was massaged with the aid of a sterile scalpel handle followed by abdomen suture. On post operatory day 1, all female rats received only water ad libitum and after they received liquid CAF diet and soft drinks. At 11 days after the RYGB and SHAM operations, the rats returned to their solid CAF diet. Thus, three groups were formed: CTL (n = 13), CAF-SHAM (n = 14) and CAF-RYGB (n = 20).

### Mating period and offspring groups

**Figure 7:**
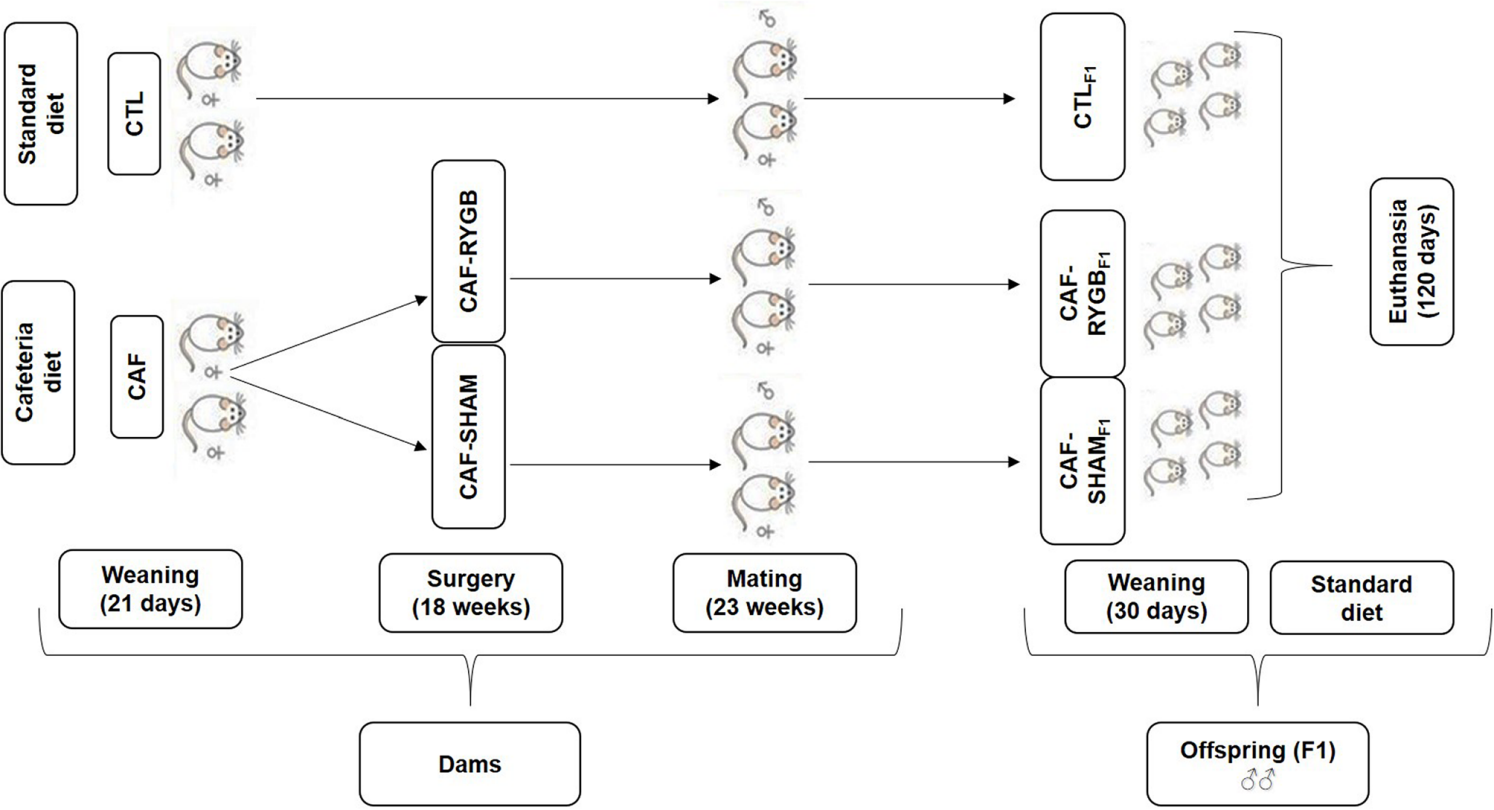
Design of experimental groups. Control Group (CTL), dams received standard diet throughout life (n = 13); Cafeteria Group (CAF), dams received cafeteria diet throughout life (n = 34); Cafeteria Group SHAM (CAF-SHAM; n = 14), dams received cafeteria diet throughout life and were submitted to sham operation; Cafeteria submitted to RYGB (CAF-RYGB; n = 20), dams received cafeteria diet throughout life and were submitted to roux y gastric bypass. Offspring (F1): CTL_F1_, offspring of rats fed on standard diet; CAF-SHAM_F1_, offspring of rats fed on CAF diet and submitted to sham operation; CAF-RYGB_F1_, offspring of rats fed on CAF diet and submitted to RYGB (n = 6–12 rats/groups). **♀** Female; ♂ male.

At five weeks after the RYGB or SHAM operations, with their respective diets, all female rats were placed in cages with sexually active control adult male rats (2 females/1 male). After pregnancy, the females were separated into individual cages until the birth of the pups. At birth, the number of pups in the offspring was adjusted to six male rats per mother and were weaned at 30 days of life. At 1 week after lactation withdrawal, the dams were euthanized. The different diets were maintained during the pregnancy and lactation phases. The offspring (F1) were denominated, according to the maternal groups: CTL_F1_: Offspring from dams that consumed rodent chow diet throughout life and were not operated; CAF-SHAM_F1_: Offspring from dams that consumed the CAF diet throughout life and were submitted to SHAM surgery; CAF-RYGB_F1_: Offspring from dams that consumed the CAF diet throughout life and were submitted to RYGB. All offspring received, from 30 to 120 days of life, standard rodent diets (BioBase, Brazil) and water ad libitum, and were maintained in adequate conditions of luminosity (7:00–19:00 h) and temperature (23 ± 2 °C). The experiments were conducted according to the guidelines of the National Council for Control of Animal Experiments (CONCEA), and norms for animal care and maintenance, as recommended by The Arrive Guidelines^73^. The experimental design is shown in Fig. 7.

### Biometric and serum biochemical parameters in maternal groups

The body weights (g) of maternal groups were determined on the day of surgery (18th week) and 3 weeks after (21th week) and then measured weekly for the rest of the study. For euthanasia, maternal groups were food deprived for 8 hours. The rats were euthanized by decapitation. Total blood was collected, and serum was used measure glucose using colorimetric enzymatic kits (Lab Test, Brazil) and for insulin measurement by immunoenzymatic assay (Sigma-Aldrich Chemicals, St Louis, MO, USA). Plasma glucose (mmol/L) and insulin (μU/mL) levels were used to calculate the homeostatic model assessment of insulin resistance (HOMA-IR) using the formula: HOMA-IR = insulin (μU/mL) × glucose (mmol/L)/22.5, as previously established by Matthews et al.^74^. This measurement has also been validated for rodents^75^. The retroperitoneal and perigonadal fat pads were removed and weighed to evaluate the abdominal fat accumulation.

### Biometric parameters and adiposity in offspring groups

The body weights (g) of the offspring were determined during three phases: at birth, at weaning and at 120 days of life. The BW and food consumption (g) were registered weekly in the offspring groups from 30 to 120 days of age. Thus, feeding efficiency [food consumption / body weight (g/g)], energy intake [food consumption × kcal/g of food (kcal)]^76^, and energy expenditure (Δ somatic energy content = total energy intake − total energy expenditure)^77^ was calculated. At 120 days of life, after a 12-h fast, the naso-anal length (NAL; cm) was measured and the Lee Index [(∛ (body weight)/NAL) × 1000] was calculated^78^. After euthanasia, the abdominal wall was laparotomized and the retroperitoneal WAT depot (WAT-R) was excised and weighed. The interscapular BAT was also excised and weighed.

### Removal of brown adipose tissue

The interscapular BAT is distributed subcutaneously between the shoulders. For anatomical reference, see a representative photo from axial cryosection in Hu et al.^79^. For removal BAT, the rats were placed with its back facing up, the dorsal coat was shaved, and a midline incision in the skin along the neck was made to expose the scapulae. The BAT was located directly under the skin between the shoulders (interscapular); it has the appearance of two lobes, butterfly-shaped, with a thin layer of white fat, that could be distinguished by the naked eye, which was carefully removed. The distinction between BAT and WAT is visible due to the large number of mitochondria that contain iron present in the BAT, providing brown colour^80^. The anterior and dorsal lobes of the interscapular brown adipose mass were located and excised, weighed, cleaned and, subsequently, the two lobes were separated. One lobe was immersed in fixative solution (4% paraformaldehyde) and used for histological analysis, while the other lobe was transferred to RNAlater solution and preserved in a freezer (−80 °C) for the Western Blotting (WB) technique.

### Histological and image analyses of BAT

After 24 h in fixative solution, the BAT fragment was washed, dehydrated in increasing alcohol solutions, diaphanized in xylol and immersed in paraffin (Paraffin Wax). The tissues were sectioned into 5-µm sections using a Reichert Jung rotary microtome (Leica RM 2025 Microsystems Inc., Wetzlar, Germany) and haematoxylin and eosin (H&E) were used for staining. Microscopic analysis of the stained preparations was performed using an Olympus BX51 (Olympus microscope, Japan) and digital photographs were taken with a 36-bit 1280 × 1024 pixel colour digital camera with a DP71 controller (Olympus). Image-Pro Plus (Media Cybernetics, Inc.) was employed for analysis of the number of nuclei (in 15.000 µm^2^) and size of adipocytes (µm^2^). Approximately 3–5 microscopic fields per section and three sections per animal (6 rats per group) were analysed. In addition, the images were treated with the aforementioned software tools, estimating the percentage (%) of the total area occupied by nuclei, lipid droplets and other components, which were grouped into the category of blood vessels and mitochondria (BVM).

### Western blotting

The BAT fragment was thawed, washed in phosphate buffer solution (PBS) and immediately homogenized for total protein dosage by the Bradford method^81^. The samples were electrophoresed in SDS PAGE (10%), transferred to nitrocellulose membrane (BioRad Laboratories) and blocked with TBS, 5% albumin for 1 h at room temperature. The nitrocellulose membrane was incubated for 12 h at 4 °C with primary antibody: OXPHOS (#ms604; Abcam) or UCP1 (#14670; Cell Signalling Technology) (1: 1000 dilutions). Antibodies against α-Tubulin (#3873; Cell Signalling Technology) or GAPDH (#g9545; Sigma Aldrich) were used as internal controls. The membrane was then washed and incubated with secondary antibody; antibody dilution was performed according to the manufacturer's instructions in TRIS-Tween buffer containing 30 g/L dry skimmed milk. Band detection was performed by chemiluminescence (Pierce Biotechnology, Rockford, IL) after incubation with horseradish peroxidase-conjugated secondary antibody. Band intensities were quantified by optical densitometry (Image J, National Institutes of Health, USA). Four rats per group were used for Western blotting analysis of all proteins tested.

### Statistical analysis

Data were analysed by Cook's distance. Normality was verified by Shapiro–Wilk tests and homoscedasticity by the Levene test. Parametric data were evaluated by analysis of variance (ANOVA) with Bonferroni post hoc (*p* < 0.05) and presented as means ± standard error of the sample mean (SEM). Nonparametric data were analysed by the Kruskall-Wallis test and Dunn's test (*p* < 0.05) and presented with median and 25th and 75th percentiles. Correlation was analysed using nonparametric Spearman’s test. R Core Team (2018—www.R-project.org) and SPSS (PASW Statistics for Windows, version 18.0. Chicago) were used for statistical analysis and Graph Pad Prism (Prism version 6.00 for Windows, La Jolla California USA, www.graphpad.com) to display the graphs.

## Supplementary Information

Supplementary Information.
