# Supplementary material for: Maternal Roux-en-Y gastric bypass surgery reduces lipid deposition and increases UCP1 expression in the brown adipose tissue of male offspring
Source: Sci Rep. 2021 Jan 13;11:1158. doi: 10.1038/s41598-020-80104-8 (PMC7806700; doi:10.1038/s41598-020-80104-8)
Supplement: Supplementary file 1 — Supplementary Information. [file 41598_2020_80104_MOESM1_ESM.pdf]

Maternal Roux-en-Y gastric bypass surgery reduces lipid deposition and increases UCP1 expression in the brown adipose tissue of male offspring

Vanessa Marieli Ceglarek*[1,4]; Iala Milene Bertasso[1]; Carla Bruna Pietrobon[1]; Nayara Carvalho Leite[2], Maria Lúcia Bonfleur[1]; Allan Cezar Faria Araújo [1]; Sandra Lucinei Balbo[1]; Sofia Pizzato Scomazzon[3]; Sabrina Grassiolli[1].

[1]Laboratory of Endocrine and Metabolic Physiology, Biosciences and Health Post-Graduate. University of West Parana, Cascavel - PR, Brazil.
[2]Obesity Comorbidities and Research Center, University of Campinas, Campinas - SP, Brazil.
[3]Medical Sciences: Endocrinology Post Graduate Program, Federal University of Rio Grande do Sul, Porto Alegre – RS, Brazil.
[4]Institute of Basic Health Sciences, Department of Physiology, Federal University of Rio Grande do Sul, Porto Alegre – RS, Brazil.

*E-mail: vanessa.ceglarek@hotmail.com

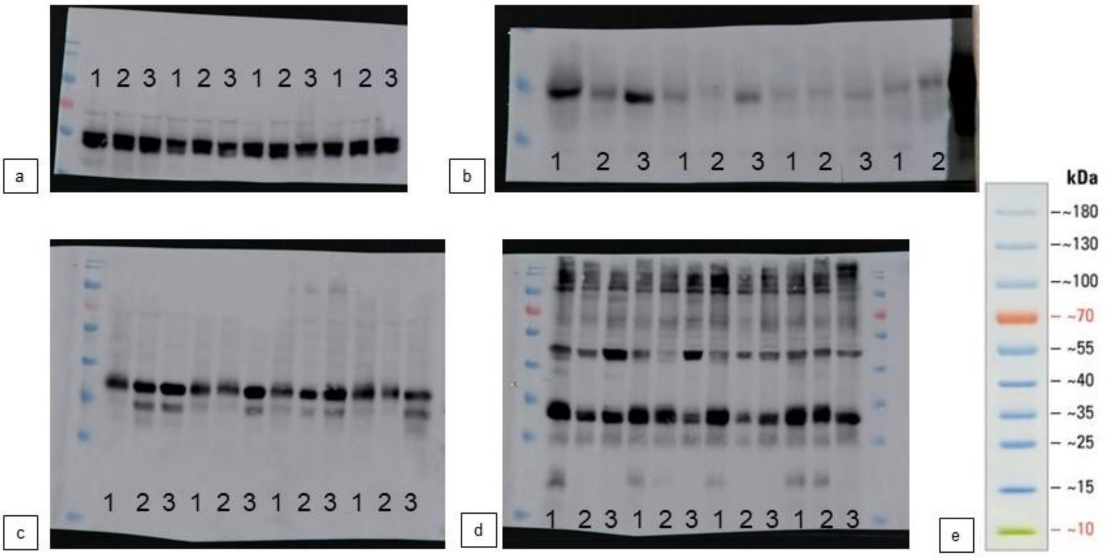

Supplementary Figure 1: Electrophoretic original images gels and blots.
1: CTL$_{F1}$; 2: CAF-SHAM$_{F1}$; 3: CAF-RYGB$_{F1}$. a: Tubulin; b: UCP1; c: GAPDH; d: OXPHOS; e: SDS-PAGE band profile of the PageRuler Prestained Protein Ladder (Thermo Scientific, #26616). All antibodies are well characterized in literature. For more information, please see manufactors datasheet.

Supplementary Table 1: Composition of food available to the animals in the cafeteria diet group.

	Energy (kcal/100 g)	Carbohydrates (g/100 g) Eq. kcal (%)	Protein (g/100 g) Eq. kcal (%)	Fat (g/100 g) Eq. kcal (%)	Na (mg/100 g)
Standard feed (BioBase, Brazil)	1591	(70%)	(20%)	(10%)	200
Italian salami (Sadia, Brazil)	362	4 (4%)	27.5 (29%)	36.25 (66%)	1621.5
Bisnaguinha (bread) (Nutrella, Brazil)	332	64 (76%)	6.8 (8%)	6 (16%)	356
Corn chips (Cheetos, Pepsico, Brazil)	483	68 (56%)	6 (5%)	20.8 (39%)	632
Marshmallow (Fini, Brazil)	330	80 (94%)	5 (6%)	0 (0%)	47.5
Sausage mixed (Sadia, Brazil)	262	0 (0%)	18 (27%)	22 (73%)	1050
Chocolate cake (Renata, Selmi, Brazil)	435	55 (52%)	5 (5%)	20 (43%)	117.5
Cornstarch cookie (Zadimel, Brazil)	436	75 (67%)	8.7 (8%)	12.3 (25%)	286.6
Mortadella (Frimesa, Brazil)	257	8.75 (14%)	13.5 (21%)	18.5 (65%)	2505
Bacon snack (Santa Helena, Brazil)	499	52 (42%)	10.5 (8%)	27.5 (50%)	1222.5
Chocolate waffer (Bauduco, Brazil)	456	53.3 (47%)	4.66 (4%)	25 (49%)	113
Soda/Coca-Cola (Coca-Cola, Brazil)	42.5	11 (100%)	0 (0%)	0 (0%)	6
Soda/Guarana (Antarctica, AmBev, Brazil)	40	10 (100%)	0 (0%)	0 (0%)	5.5

Kcal, kilocalories. g, grams. Eq, equivalent. To convert kilocalories into kilojoules, multiply by 4.19.
